# Investigation of the Flammability and Thermal Stability of Halogen-Free Intumescent System in Biopolymer Composites Containing Biobased Carbonization Agent and Mechanism of Their Char Formation

**DOI:** 10.3390/polym11010048

**Published:** 2018-12-30

**Authors:** Muhammad Maqsood, Gunnar Seide

**Affiliations:** Faculty of Science and Engineering, Maastricht University, Urmonderbaan 22, 6167 RD Geleen, The Netherlands; gunnar.seide@maastrichtuniversity.nl

**Keywords:** biopolymer composites, carbonization agent, thermal analysis, flame retardancy, char formation

## Abstract

Starch, being a polyhydric compound with its natural charring ability, is an ideal candidate to serve as a carbonization agent in an intumescent system. This charring ability of starch, if accompanied by an acidic source, can generate an effective intumescent flame retardant (IFR) system, but the performance of starch-based composites in an IFR system has not been tested in detail. Here, we describe a PLA-based IFR system consisting of ammonium polyphosphate (APP) as acidic source and cornstarch as carbon source. We prepared different formulations by melt compounding followed by molding into sheets by hot pressing. The thermal behavior and surface morphology of the composites was investigated by thermogravimetric analysis and scanning electron microscopy respectively. We also conducted limiting oxygen index (LOI), UL-94, and cone calorimetry tests to characterize the flame-retardant properties. Cone calorimetry revealed a 66% reduction in the peak heat release rate of the IFR composites compared to pure PLA and indicated the development of an intumescent structure by leaving a residual mass of 43% relative to the initial mass of the sample. A mechanism of char formation has also been discussed in detail.

## 1. Introduction

Biobased polymers are derived from renewable resources, and their importance has grown over the last decade because they address current challenges such as the depletion of petroleum reserves (the feedstock for conventional plastics) and the environmental harm caused by the irresponsible disposal of non-degradable polymers [1]. One of the most widely used biobased polymers is polylactic acid (PLA), a thermoplastic polymer obtained from renewable resources such as corn starch [2]. PLA is less flammable than synthetic thermoplastics and produces less visible smoke when burning, resulting in a lower peak heat release rate than polyethylene terephthalate (PET) [3]. Even so, PLA is still combustible, which limits its applications in the automobile, electrical, and electronics sectors and in the production of flame-retardant materials [4].

Intumescent flame retardant (IFR) systems offer a highly effective strategy to enhance the fire retardancy of PLA because a char structure is developed that acts as a shield between the polymer and heat source, hence protecting the polymer material from further burning and dripping [5,6]. Modern IFR systems are based on halogen-free flame retardants (HFFRs) which, unlike their halogen-containing counterparts, are most of the time environmentally safe [6]. Furthermore, HFFRs are not only environmentally safe but also extremely efficient [7]. IFR system typically comprise three constituents: an acidic source, a blowing agent, and carbonic source to produce a char layer [8].

In previous studies various attempts have been made in order to improve the flame retardancy of PLA by using different formulations and additive types such as nitrogen-based compounds [9], phosphorous-based compounds [10], silicon-based compounds [11], expanded graphite– or carbon-based compounds [12], halogen-containing compounds [13,14], and halogen-free compounds as flame retardants [15,16]. However, intumescent flame retardants (IFRs) containing an acidic and carbonic source have proven to be the most effective [17,18]. Traditional IFR systems often contain ammonium polyphosphate (APP) as the acidic source, melamine (MEL) as the blowing agent, and pentaerythritol (PER) as the carbonic source [16]. The thermal degradation of phosphorous-comprising fire retardants such as APP results in the formation of pyrophosphate and the release water, which eventually dilutes the gas phase such that the dehydration reaction is catalysed by pyro-phosphoric acid [19,20]. Various alternative formulations have also been tested including PLA-based composites containing spirocyclic PER bisphosphorate disphosphoryl melamine [17] and microcellular PLA composite foams with graphene as the carbonization agent [18].

The importance of IFRs containing biopolymers with biobased carbonic source and halogen free acidic source has grown interest throughout the last decade in order to promote the sustainable approach toward flame retardancy of polymers [4]. Therefore, in continuation to this approach various researchers tried different formulations in IFR systems with different halogen-free acidic sources such as phytic acid [19], fumaric acid [20], and biobased carbonic sources such as cyclodextrin [21], sorbitol [22], chitosan [20], and lignin [1]. Conventional carbonization agents are effective in some polyolefin-based IFR systems [5] but are not compatible with PLA [18]. For example, although PER combined with APP resulted in a substantial progress in flame retardancy, the composites achieved only a V-2 rating in the UL94 test despite of the addition of 30–40% *w*/*w* of the additive [9]. Although char promoting effect of starch has been investigated in some studies [23,24], those studies did not describe the mechanism of char formation, and the additive content (wt %) in those studies [25,26] were too high to achieve the desired results.

We therefore investigated the effect of cornstarch (ST) as a carbonization agent in IFR systems as a potential biobased substitute for PER together with non-toxic and halogen-free flame retardant and studied the mechanism of char formation. The mechanism of intumescence indicating catalytic phosphorylation to produce phosphate esters, which eventually dehydrated the starch and formed char structure containing residue up to 43% has also been discussed in detail. We prepared PLA-based IFR systems containing different amounts of APP or APP together with starch by melt compounding and then molding the composites into sheets by hot pressing. Thermogravimetric analysis (TGA) was done to test the thermal behavior whereas scanning electron microscopy was used to determine the surface morphology of the composites. The limiting oxygen index and UL-94 vertical burning tests were conducted to determine the flame-retardant properties of the composites, whereas char residues were characterized by cone calorimetry test.

## 2. Materials and Methods

### 2.1. Materials

PLA polymer in granule form was obtained from Total|Corbion (Gorinchem, The Netherlands). Non-halogenated flame retardant Exolit AP 422, a fine-particle ammonium polyphosphate (APP) containing 14% (*w*/*w*) nitrogen and 31% (*w*/*w*) phosphorous, was obtained from Clariant (Muttenz, Switzerland). The decomposition temperature of Exolit AP 422 was higher than 275 °C. Corn-based starch (particle size 100 μm) was obtained from Royal Ingredients Group B.V. (Alkmaar, The Netherlands). PLA, APP, and starch were vacuum-dried at 80 °C for 6 h before use.

### 2.2. Preparation of PLA/IFR Composites

PLA/APP and PLA/APP/ST composites were prepared using a KETSE 20/40 compounder (Brabender, Duisburg, Germany) at 190 °C. Initially, PLA/APP composites containing 10%, 15%, or 20% (*w*/*w*) APP (hereafter PLA/APP10, PLA/APP15, and PLA/APP20) were compounded at a screw rotation speed of 150 rpm. The temperatures of the three heating zones were kept at 180, 185, and 190 °C, respectively. The extrudate was cut in to pellets. We also modified the PLA/APP20 pellets to incorporate 3%, 5%, or 7% (*w*/*w*) corn starch (hereafter PLA/APP20/ST3, PLA/APP20/ST5, and PLA/APP20/ST7) at a screw rotation speed of 200 rpm. PLA/APP20 pellets were incorporated in the first feeding zone whereas starch was added in the second feeding zone. This procedure was used to ensure proper mixing of all the components. Sheets of the prepared composites (100 × 100 × 3 mm^3^) were produced by compression molding at 190 °C. Sheets of pure PLA were also prepared with the same dimensions for comparison. Sheets were cut in to different specimens as per the requirement of each fire test. The formulations of the as-prepared composites are summarized in Table 1.

### 2.3. Mechanism of Char Formation

**Figure 1:**
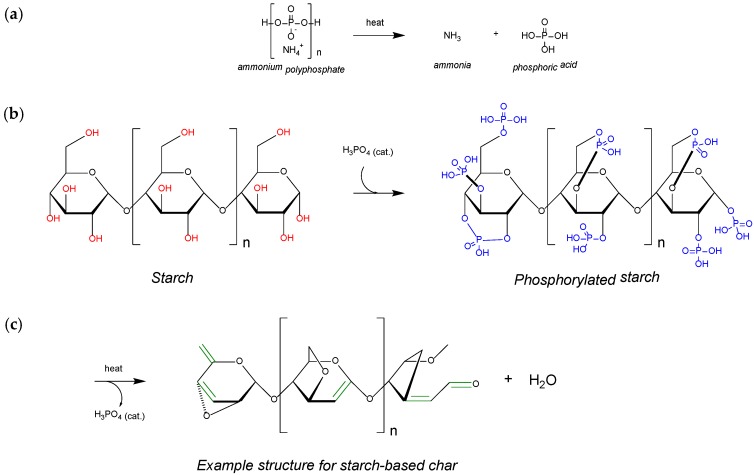
Thermal decomposition of ammonium polyphosphate into ammonia and ortho-phosphoric acid (**a**), Catalytic phosphorylation to produce phosphate esters (**b**), Dehydration of starch and formation of starch-based char structure (**c**).

Long chain APP (Form II) was used as flame retardant in PLA polymer. Upon decomposition of APP, phosphoric acid and ammonia was formed. Phosphoric acid acted as acid catalyst in the dehydration process of hydroxyl groups in starch. Upon reaction of acid catalyst (phosphoric acid) with hydroxyl groups in starch, phosphate esters were formed that were decomposed later to release carbon dioxide, and dehydration of starch took place. In the gas phase, the emission of carbon dioxide helped in dilution of the oxygen present in air together with the by-products that were ignited during decomposition of the materials, whereas the resultant char layer in the condensed phase protected the underlying polymeric material from further burning by restricting the free passage of radiant heat and oxygen. This mechanism of intumescence is shown in Figure 1.

### 2.4. Limiting Oxygen Index and UL-94 Vertical Burning Test

The limiting oxygen index (LOI) is the fraction of oxygen that must be present to support burning, hence higher LOI values indicate lower flammability. The specimens (100 × 10 × 3 mm^3^, as required by ISO 4589) were vertically placed in a glass column supplied with a mixture of oxygen and nitrogen gas and were then ignited from above using a downward-pointing flame. The LOI test was conducted using a Stanton Redcroft instrument (Illinois Toolworks, Glenview, IL, USA).

The UL-94 test classifies materials based on their ability to either promote or inhibit the spread of fire once it has been ignited. UL-94 tests were conducted using specimens with dimensions of 100 × 10 × 3 mm^3^ as required by ISO 9773. A flame was applied to the bottom of a vertically supported specimen, and the response was assessed after removing the flame. Specimens that self-extinguish and do not drip after burning are ranked highest in the classification (V-0).

### 2.5. Cone Calorimetry Test

Cone calorimetry works on the principle of oxygen consumption and states that the total heat of combustion of a specimen depends on the quantity of oxygen consumed. The cone calorimeter tests were conducted on specimens with dimensions of 100 × 100 × 3 mm^3^ as required by ISO 5660 using a Stanton Redcroft instrument. The samples were exposed to a heat flux of 35 kW m^−2^. We then recorded the heat release rate (HRR), total heat release rate (THRR), time to ignition (TTI), and percentage mass residue after burning.

### 2.6. Thermogravimetric Analysis

Thermogravimetric analysis (TGA) was conducted using a Q5000 device (TA Instruments, New Castle, DE, USA). The specimens (4–5 mg) were heated at a constant rate of 10 °C min^−1^ up to 700 °C under nitrogen at a flow rate of 50 mL min^−1^. The thermal degradation temperature and the temperature at which maximum degradation took place were calculated along with the residual percentage mass of the sample and TGA curves were plotted for each specimen.

### 2.7. Scanning Electron Microscopy

The surface morphology, dispersion of FR additives in the PLA matrix and char residues of PLA/APP and PLA/APP/ST composites were investigated by scanning electron microscopy (SEM) using a TM-1000 table-top microscope (Hitachi, Chiyoda, Tokyo, Japan). The samples were immersed in liquid nitrogen followed by freeze fracturing and gold sputtering to produce a conductive surface.

### 2.8. Mechanical Testing

Mechanical properties such as tensile strength, elongation at break and Young’s modulus of PLA, PLA/APP and PLA/APP/ST composites were tested by Zwick Roell Z020TH allround-line table-top machine (Zwick GmbH & Co.KG, Ulm, Germany) at a speed of 50 mm min^−1^. The test specimens of dog bone shape were prepared as per standard EN ISO 527-2 method using a molding press. Six specimens were prepared from each formulation, and their average results with standard deviations were recorded. Specimens dimensions used were 170 × 20 × 3 mm^3^.

## 3. Results and Discussion

### 3.1. LOI and UL-94 Vertical Burning Test

The LOI and UL-94 tests are widely accepted to assess the flame retardancy of FR composites and the corresponding results (including dripping behavior) for the PLA/APP and PLA/APP/ST composites are summarized in Table 1.

Pure PLA did not pass UL-94 test because it was highly flammable with prolific dripping, and the LOI was 19.5%. The addition of 10 wt % APP (PLA/APP10) increased the LOI to 24.4% and the composite achieved a V-2 rating in UL-94 test. The presence of 15 and 20 wt % APP (PLA/APP15 and PLA/APP/20) increased the LOI to 28.5% and 31.9%, respectively, and both composites obtained a V-1 and V-0 rating in UL-94 test respectively. Even so, both of these composites showed some dripping behavior during second flame application.

With the addition of 3 wt % ST (PLA/APP20/ST3), the LOI increased from 31.9% to 34.5%, and the composite retained its V-0 rating in the UL-94 test. There was also no evidence of dripping during first and subsequent test. Higher concentrations of ST (PLA/APP20/ST5 and PLA/APP20/ST7) increased the LOI to 36.2% and 37.3%, respectively, and both composites obtained V-0 classification in the UL-94 test. In this case, however, there was no dripping during either the first or the second test.

The char structure sheltered the underlying material from the external heat source, pyrolysis gases as well as from thermal degradation therefore the ignition process was either delayed or prevented hence enhancing the flame retardancy of starch-based composites. The formation of char is ascribed by the decarboxylation and dehydration reactions caused by the catalytic effect of starch in PLA/APP/ST composites. These results confirmed that the introduction of ST as a natural carbonization agent increased the LOI values of the FR composites significantly while simultaneously inhibiting the melt dripping phenomenon. All composites containing ST managed to obtain a V-0 rating in the UL-94 test. Compared to a similar study done by Marosi et al. [25], where they achieved LOI value of 34% by adding up to 11 wt % of starch, we managed to achieve LOI value of 37.3% by adding only 7 wt % of starch.

The addition of APP in the concentration range of 10% (*w*/*w*) to 20% (*w*/*w*) enhanced the LOI from 24.4% (PLA/APP10) to 31.9% (PLA/APP20) (Table 1). By increasing the amount of APP, a higher concentration of oxygen is needed to achieve the ignition of the sample due to the dilution of the fuel in the gas phase by the discharge of water vapor as a result of the dehydration of APP. The addition of ST to the formulations not only increased the LOI of the samples but also increased the mass residue, providing enhanced shielding against heat and a barrier against the emission of pyrolysis gases that act as fuel. Therefore, the emission of fuel in the gas phase is minimized by the addition of ST.

**Figure 2:**
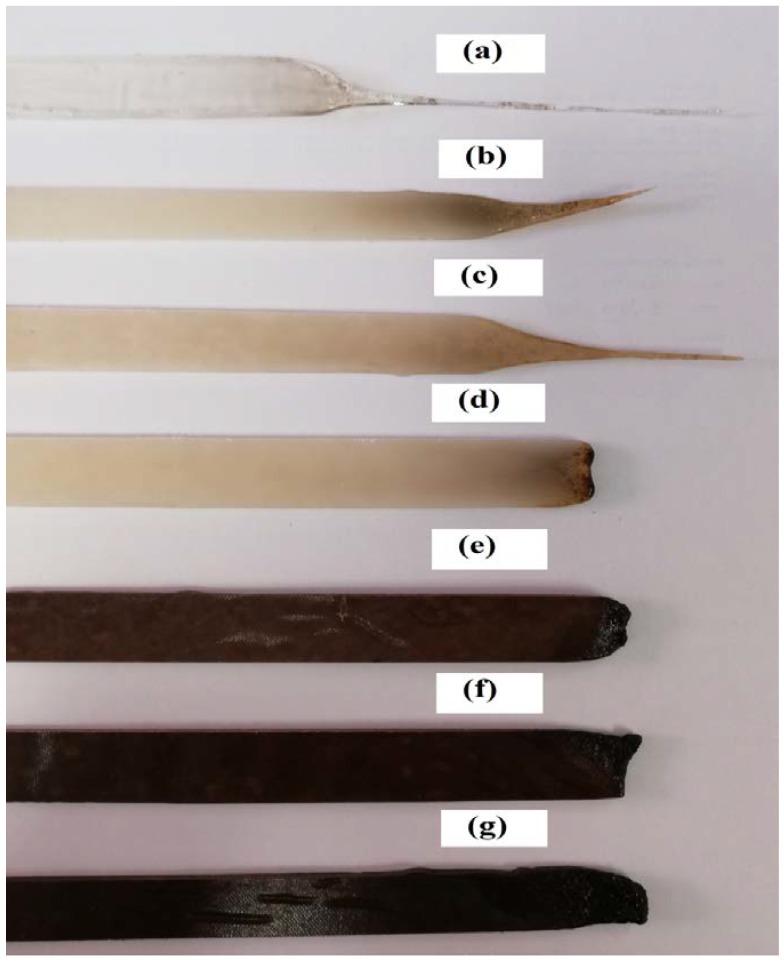
Photographs of pure PLA (**a**), PLA/APP10 (**b**), PLA/APP15 (**c**), PLA/APP20 (**d**), PLA/APP20/ST3 (**e**), PLA/APP20/ST5 (**f**), and PLA/APP20/ST7 (**g**) composites after UL-94 test.

The UL-94 test classifies materials based on their ability to either promote or inhibit the spread of fire once it has been ignited. Pure PLA ignited during the first flame application (10 s), and the sample continued to burn until it was fully consumed. Although PLA/APP10 and PLA/APP15 performed better as flame retardants (flame extinguished less than 30 s after each flame application; V-2 and V-1 ratings, respectively), the dripping of the burning sample ignited the cotton placed beneath. Similarly, PLA/APP20 achieved a V-0 rating because the flame was extinguished in less than 10 s, but these samples still showed dripping behavior during second flame application. In contrast, none of the composites containing starch were dripping even after the second application of flame and all achieved a V-0 rating due to the generation of char layer on the surface which isolated the remaining sample and prevented the propagation of the flame. In previous studies [9,27], even the addition of 30–40% (*w*/*w*) PER as a carbonization agent was sufficient to achieve only a V-2 rating, whereas here we found that as little as 3% starch in the presence of 20% APP accomplished the target rating of V-0. Compared to another similar study done by Casetta et al. [24] where they achieved V-0 rating in UL-94 vertical burning test by adding up to 10 wt % of starch together with 30 wt % of APP, whereas in our results we managed to achieve V-0 rating in UL-94 vertical burning test by adding only 3 wt % of starch together with 20 wt % of APP. Hence, these results are in good relation with the main hypothesis of this study. The photographs of the test samples after UL-94 test are shown in Figure 2, which confirms the formation of char layer after burning on samples surface containing starch as carbonization agent.

### 3.2. Cone Calorimetry

Cone calorimetry provides broad information about the combustion behavior of polymers by measuring parameters such as time to ignition (TTI), peak heat release rate (PHRR), and total heat release rate (THR), which can predict their behavior in real-life fires.

**Figure 3:**
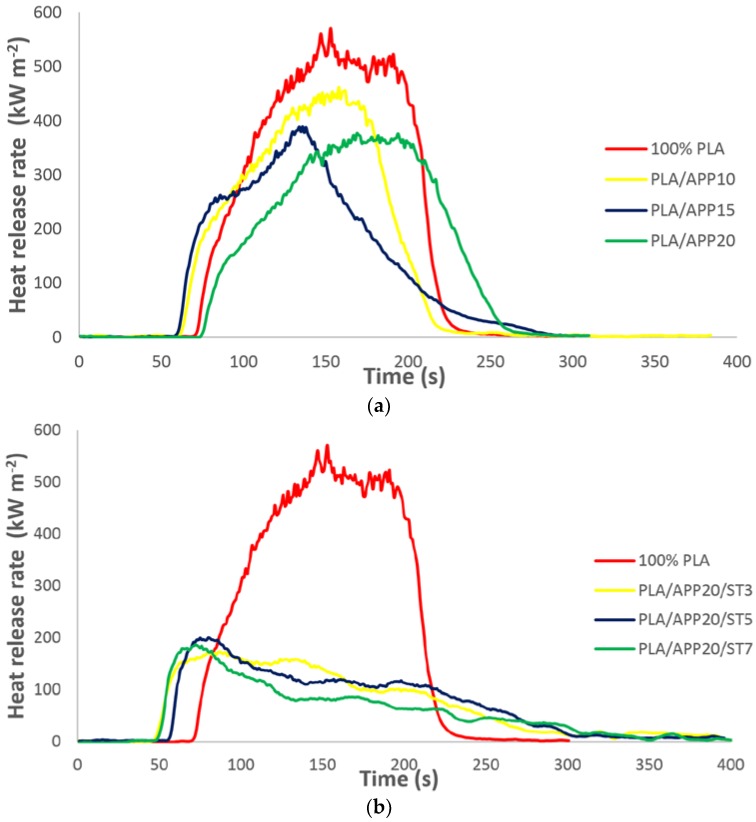
(**a**) Heat release rate curves of pure PLA and PLA/APP composites. (**b**) Heat release rate curves of pure PLA and PLA/APP/ST composites.

The heat release rate (HRR) curves of pure PLA, PLA/APP10, PLA/APP15, PLA/APP20, PLA/APP20/ST3, PLA/APP20/ST5, and PLA/APP20/ST7 are presented in Figure 3a,b. Following ignition, pure PLA burnt much faster than the other samples and produced a very sharp HRR curve with a PHRR of 570 kW m^−2^. For composite PLA/APP20, the PHRR declined to 337 kW m^−2^, and with the further addition of 3 wt % ST (PLA/APP20/ST3), the PHRR was even lower, at 212 kW m^−2^. At the maximum 7 wt % ST content we tested (PLA/APP20/ST7), the PHRR was only 192 kW m^−2^, which is 66.30% of the pure PLA value. These findings indicated that the combined effect of APP and ST allowed the formation of a much thicker char layer on the surface of the composites after ignition, which prevented the degradation of the composite by restricting the fire passage into the polymer matrix.

In IFR systems, flame retardancy is achieved by the swelling of the substrate in the condensed phase, which generates a sponge-like multicellular structure called char that shelters the principal material from heat transfer. The char structure also acts as a physical barrier against fuel and mass transfer from the condensed phase to the site of burning. Figure 3a,b demonstrates that the heat release rate of the composites containing APP alone (PLA/APP10, PLA/APP15, and PLA/APP20) or together with starch (PLA/APP20/ST3, PLA/APP20/ST5, and PLA/APP20/ST7) changed dramatically in comparison to pure PLA (570 kW m^−2^). In samples containing APP alone, the intumescent char layer was thinner and more porous than in samples containing APP and ST. This is because the absence of ST lowered the viscosity of the char layer, in turn allowing vapor and gas bubbles to escape and reducing the degree of swelling because little pressure was allowed to build up. The resulting porous structure allowed further fuel gases and water vapor to pass through the unclosed cells, increasing the PHRR. In contrast, the higher viscosity of the char layer containing ST made the char more compact and prevented the escape of gases and vapor, resulting in a pressure build up that increased the melt viscosity of the condensed phase and resulted in more swelling of the char. The combined effect of APP and ST therefore reduced the PHRR to 192 kW m^−2^, which is 66.30% less than pure PLA. The HRR in this study is much lower than reported in other studies of PLA composites containing different carbonization agents [28,29,30].

Table 2 shows that the TTI of pure PLA was 41 s, increasing to 58 s when APP was incorporated into the PLA matrix (PLA/APP20) and to 77 s when the maximum content of starch was included (PLA/APP20/ST7). The ignition of a material is normally dependent on the concentration of pyrolysis gases, which are released when a material is degraded. The concentration of the gases increases during material degradation and ignition starts when they reach a certain threshold. Longer ignition times reflect the slower decomposition of the material mainly due to the presence of starch together with APP. A uniform and compact char structure can hinder the diffusion of pyrolysis gases from the melting substrate to the site of burning. The lower TTI of the samples containing APP alone is mainly due to the emission of more pyrolysis gases, reflecting the weaker swelling of the substrate and the generation of a porous structure as discussed above. The TTI is therefore increased by the more compact char structure in the PLA/APP/ST composites.

**Figure 4:**
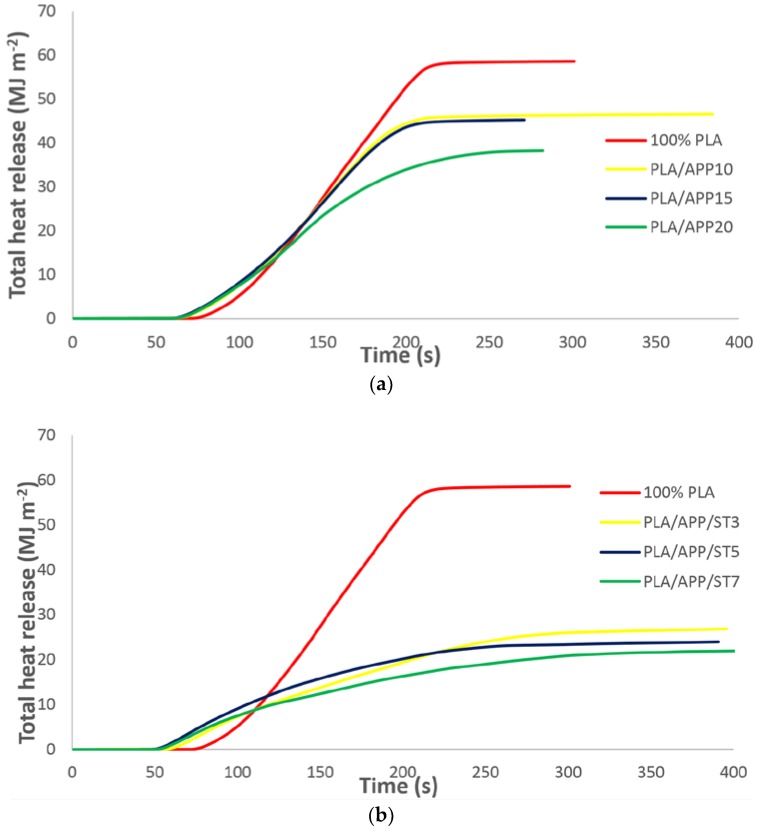
(**a**) Total heat release curves of pure PLA and PLA/APP composites. (**b**) Total heat release curves of pure PLA and PLA/APP/ST composites.

Figure 4a,b shows the THR curves of pure PLA and the PLA composites. Figure 4a indicates that the THR of pure PLA was 58 MJ m^−2^ whereas the PLA/APP20 and PLA/APP20/ST7 composites emitted only 37 and 24 MJ m^−2^, respectively. The PLA/APP20 and PLA/APP20/ST7 composites therefore limited the total amount of fuel accessible for burning, which confirms the superior FR properties of these composites. The combination of starch and APP makes the composites more flame resistant. The formation of intumescent char on matrix surface improves the thermal insulation between the flame and material’s surface. This extinguishes the flame by preventing access to combustible gases and oxygen at the site of the fire.

The combination of ST and APP makes the composites more flame resistant, and the formation of intumescent char on the matrix surface introduced a layer of thermal insulation between the flame and the surface of the material, which extinguished the flame by preventing contact with combustible gases as well as oxygen. The high concentrations of APP and ST diluted the polymer matrix, providing less material for continued burning. Thermal decomposition therefore led to the dehydration of APP, and the resulting water vapors cooled the gas phase and diluted the fuel, thus reducing the total heat release (THR) in proportion with the increasing APP content. Due to the endothermic decomposition of APP, the heating of the condensed phase was also limited. The presence of ST exacerbated this effect because the emission of pyrolysis gases was inhibited by the formation of the char layer, which provided a physical barrier and enhanced the heat shielding effect. In previous studies involving PLA composites with other carbonization agents, the THR was much higher than the values reported here [11,31,32].

**Figure 5:**
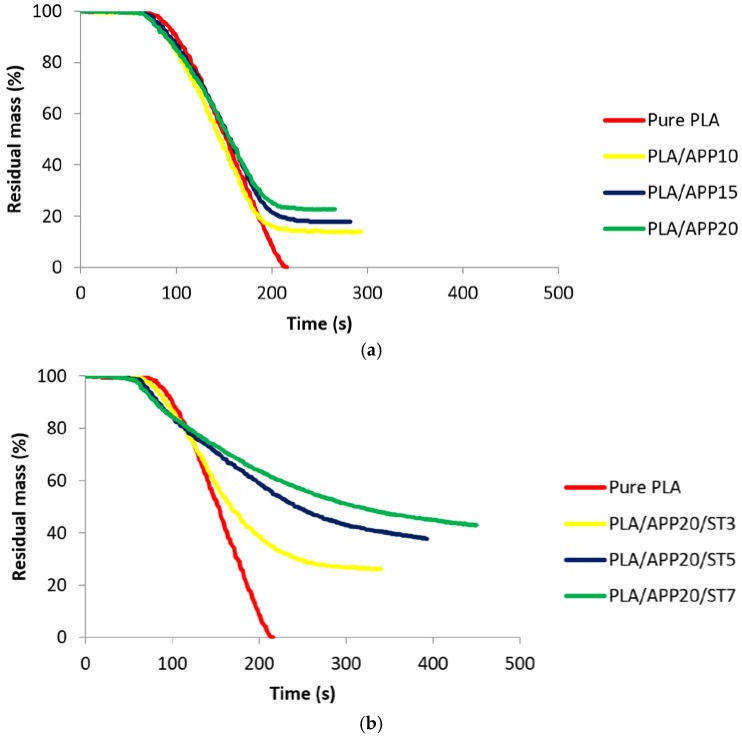
(**a**) Residual mass% of pure PLA and PLA/APP composites. (**b**) Residual mass% of pure PLA and PLA/APP/ST composites.

Figure 5a,b shows the residual mass% after burning for pure PLA, PLA/APP, and PLA/APP/ST composites. No residual mass was left following the burning of pure PLA, but both PLA/APP20 and PLA/APP20/ST7 left mass residues corresponding to 22.74% and 43.00% of the starting mass, respectively, as shown in Table 2. The relatively large proportion of residual mass (char residue) for PLA/APP20/ST7 probably reflects the development of a nanostructure that hindered the passage of fuel and heat during combustion. The higher residual mass correlated with the production of more char, which in turn reflects the lower THR values. The greater residual mass also reflects an increase in char formation due to the combined effect of the acid and carbonization agent. The percentage residual mass achieved in this study is also higher than that reported in previous studies of PLA composites containing alternative carbonization agents [17,33].

**Figure 6:**
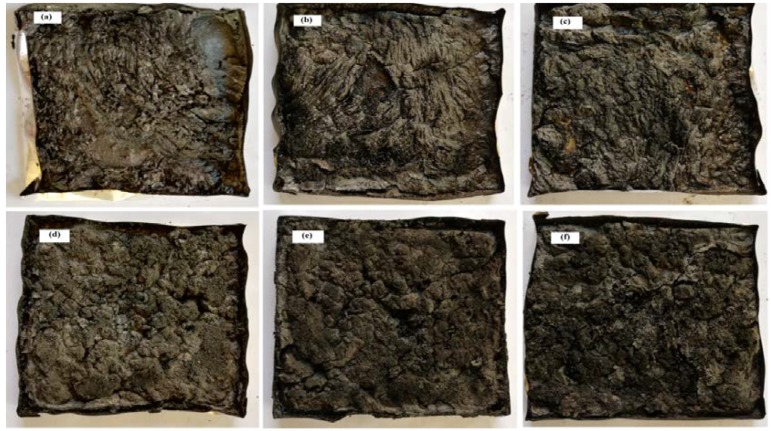
Photographs of the residues of PLA/APP10 (**a**), PLA/APP15 (**b**), PLA/APP20 (**c**), PLA/APP20/ST3 (**d**), PLA/APP20/ST5 (**e**), and PLA/APP20/ST7 (**f**) after cone calorimetry test.

**Figure 7:**
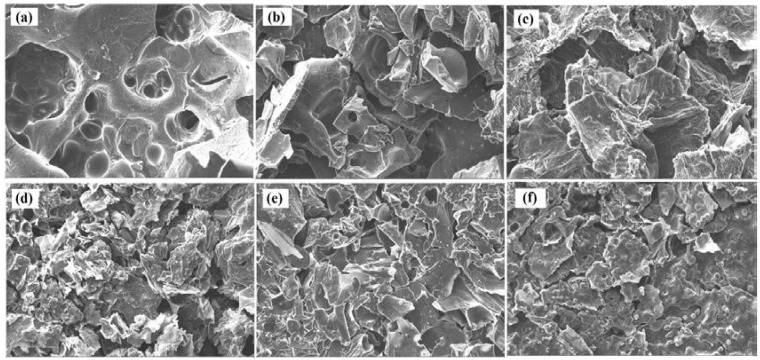
SEM analysis of the residues of PLA/APP10 (**a**), PLA/APP15 (**b**), PLA/APP20 (**c**), PLA/APP20/ST3 (**d**), PLA/APP20/ST5 (**e**), and PLA/APP20/ST7 (**f**) after cone calorimetry test. Scale bar in all panels = 10 μm, Magnification = 710×.

Figure 6 shows images of the residual samples after cone calorimetry test. As stated above, there was almost no residue of pure PLA, but the samples containing APP and starch presented intumescence with char on the surface, which was thicker and more stable in the case of PLA/APP20/ST7. The char residues of PLA/APP10, PLA/APP15 and PLA/APP20 were loosely bound due to the non-cohesion of the agglomerates, and the structure in each case was porous and discontinuous due to insufficient char formation as indicated by the SEM analysis of char residues in Figure 7. Heat and mass transfer therefore could not be inhibited effectively in these composites. In contrast, the samples containing ST (particularly PLA/APP20/ST7) produced a more compact char (Figure 7) with a dense and uniform structure, reducing the heat and mass transfer to inhibit combustion and prevent further burning of the underlying polymeric substrate. These char structures were stable, more uniform, and compact due to the cohesion of the agglomerates. ST particles were supposed to fill the empty spaces between the APP particles, with a resulting increase in density. The thickness of the samples containing ST also increased dramatically due to char formation after burning, from an initial thickness of 3 mm to approximately 1.5–2.0 cm.

### 3.3. Thermogravimetric Analysis

**Figure 8:**
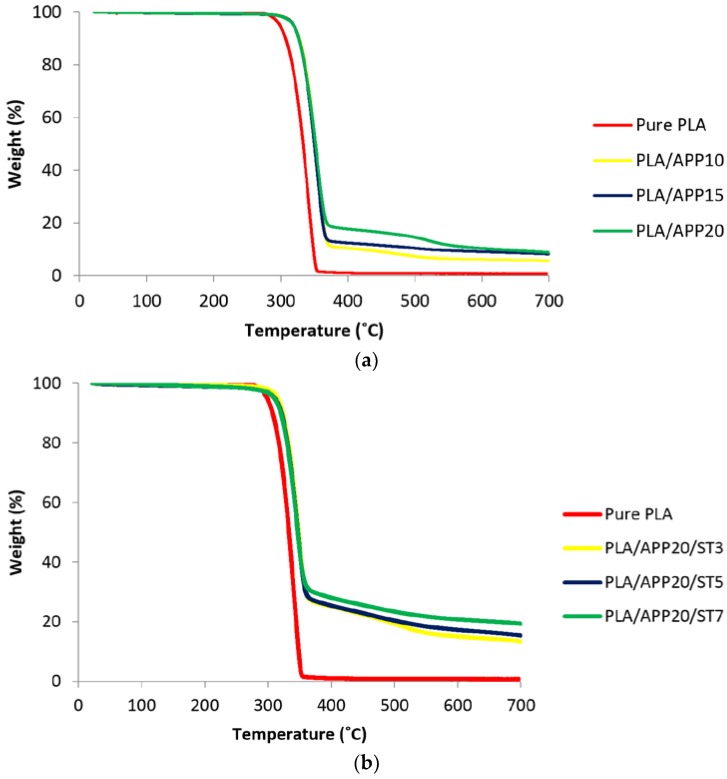
(**a**) Thermogravimetric analysis curves of pure PLA and PLA/APP composites. (**b**) Thermogravimetric analysis curves of pure PLA and PLA/APP/ST composites.

The thermal decomposition and thermal stability of polymers is most effectively assessed by thermogravimetric analysis (TGA). The thermal degradation and mass residue of the samples were compared to determine the influence of flame retardants and starch on PLA-based composites. TGA curves and data for all the composites heated in a nitrogen atmosphere are presented in Figure 8a,b and in Table 3, respectively.

In Table 3, the temperatures corresponding to 5% and 50% weight loss for each composite are represented by the T5 and T50 values, respectively, whereas the temperature corresponding to the maximum rate of weight losses is represented by T max. The degradation of pure PLA started at 325 °C and 50% loss occurred at 372 °C, with no residue left at 700 °C. A similar trend was observed for PLA/APP10 for the T5 and T50 temperatures, but the residue left at 700 °C was 5.90% of the initial mass. For PLA/APP15 and PLA/APP20, the initial decomposition temperatures and thermal stabilities were greater than the corresponding values for PLA/APP10, with 8.16% and 9.21% residual mass left at 700 °C. The introduction of starch further improved the thermal stability of the composites. The initial decomposition temperatures and thermal stabilities of all composites containing starch are higher compared to composites without starch. For example, composite PLA/APP20/ST3 increased the residual mass at 700 °C from 9.21% to 13.34%, but this increased even further to 19.30% in the case of composite PLA/APP20/ST7.

Figure 8a represents the TGA curves for PLA/APP composites in comparison to pure PLA whereas Figure 8b shows TGA curves for PLA/APP/ST composites. These composites differ in terms of their initial decomposition temperatures and thermal stabilities. The initial decomposition temperature of PLA/APP20/ST7 was 373 °C, compared to 365 °C for PLA/APP20/ST3, and the residues left at 700 °C were 19.30% and 13.34% of the initial mass, respectively. PLA/APP20/ST7 is therefore more thermally stable, reflecting the denser and more compact char layer as discussed above. These TGA data are in strong agreement with the LOI, UL-94 and cone calorimetry experiments, indicating that composites containing starch are superior in performance to composites containing APP alone.

### 3.4. SEM Analysis

**Figure 9:**
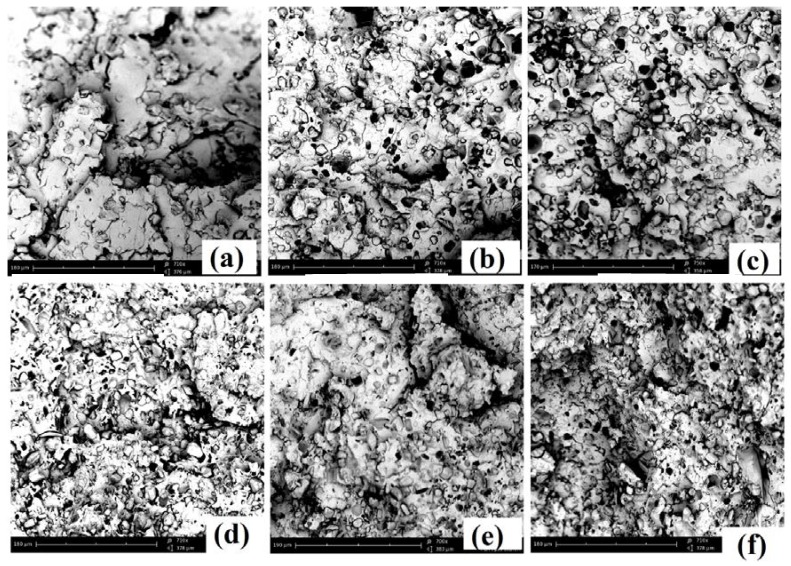
SEM analysis of PLA/APP10 (**a**), PLA/APP15 (**b**), PLA/APP20 (**c**), PLA/APP20/ST3 (**d**), PLA/APP20/ST5 (**e**), and PLA/APP20/ST7 (**f**) composites. Scale bar in all panels = 180 μm, Magnification = 710×.

Better FR properties are achieved by the uniform dispersion of additives in the PLA matrix. We therefore investigated the appearance of various percentages of FR additives (APP) and starch (ST) dispersed into the PLA matrix by scanning electron microscopy (SEM). Figure 9 shows the SEM images of the PLA/APP10 (a), PLA/APP15 (b), PLA/APP20 (c), PLA/APP20/ST3 (d), PLA/APP20/ST5 (e), and PLA/APP20/ST7 (f) composites. We observed APP and ST particles of different sizes and shapes, and with different levels of interfacial adhesion with the PLA matrix. In all formulations of PLA/APP, the FR additive was uniformly distributed. The appearance of the dispersions was similar regardless of the FR content, indicating that the additives and substrate mixed uniformly during sample preparation. However, we observed very weak interfacial bonding between the FR additive and PLA substrate as shown by the appearance of small holes during fracturing. In the PLA/APP/ST composites, the dispersion of APP and ST was less uniform compared to the PLA/APP composites because the additives were less compatible with each other in PLA matrix, therefore forming isolated or aggregated particles on the composites surfaces. These clustered and agglomerated particles were clearly seen in the SEM images. However, all images indicated that APP and ST were successfully incorporated into the PLA matrix.

### 3.5. Mechanical Testing

The mechanical properties of composites are dependent on the actual stress sharing between matrix and the additives incorporated. Therefore, in order to get better mechanical properties of a composite a uniform interfacial bonding between additives and matrix is needed. Moreover, the size of particles, wt % (*w*/*w*) of additives incorporated as well as the adhesion between additives and matrix influence the mechanical properties of polymer composites. As indicated in SEM images in the previous section a weak interfacial bonding between additives and polymer matrix was observed, due to which clustered and agglomerated particles were formed which affected the mechanical strength of the composites.

It can be seen in Table 4 that the tensile strength and elongation at break of pure PLA was 69.19 (MPa) and 2.49% respectively. However, with the addition of APP alone the tensile strength and elongation at break started to decrease and reached to 45.62 (MPa) and 1.98%, respectively, when 20 wt % of APP (PLA/APP20) was incorporated in PLA matrix. When starch was incorporated together with APP in polymer matrix (PLA/APP/ST), tensile strength and elongation at break was further reduced. The reduction in mechanical properties of PLA/APP and PLA/APP/ST composites is mainly due to weak interfacial bonding initiated by the difference in polarity among PLA matrix, APP, and starch additives.

Another reason of weaker mechanical properties could be due to the degradation of PLA as well as of starch during preparation of PLA composites due to higher extrusion temperature which might have reduced the adsorbed chains mobility on the surface of the particles. Therefore, in order to improve the mechanical properties of PLA composites a uniform dispersion of additives in polymer matrix may be required which sometimes can be obtained by the use of a compatibilizer. Although the addition of starch in polymer matrix decreased the mechanical properties of the composites, however extraordinary improvements in the flame-retardant properties of these composites were seen.

## 4. Conclusions

We have produced intumescent flame-retardant composites by combining PLA and APP with starch as a carbonization agent. PLA/APP and PLA/APP/ST composites were prepared, and their flammability was assessed by LOI, UL-94 and cone calorimetry tests. The addition of 10–20 wt % APP improved the LOI of PLA from 19.5 to 24.4–31.9%, but the further inclusion of 7 wt % starch (PLA/APP20/ST7) improved the LOI from 31.9% to 37.3% and the composite achieved a V-0 rating in the UL-94 test with no dripping. The PHRR and THR of the composites containing starch were significantly lower than the corresponding values for pure PLA and composites containing APP alone. A remarkably low PHRR was observed for PLA/APP20/ST7 (192 kW m^−2^) which is 66% less than the PHRR of pure PLA. The presence of 20 wt % APP in PLA matrix (PLA/APP20) increased the TTI to 58 s, but the addition of 7 wt % starch in addition to APP (PLA/APP20/ST7) extended this to 77 s. The THR of pure PLA was 58 MJ/m^2^, falling to 38 MJ/m^2^ for PLA/APP20 and 24 MJ/m^2^ for PLA/APP20/ST7. The composites therefore limited the total quantity of fuel accessible for burning. The introduction of APP together with starch enhanced the thermal stability of the composites, with PLA/APP20/ST7 leaving 19.30% residual mass at 700 °C comparted to only 9.21% for PLA/APP20 and no residue for pure PLA. The fire-retardant mechanism was determined by cone calorimetry, revealing that char formation inhibited the initial decomposition of the composite and improved its thermal stability by creating a char layer which prevented the transfer of sufficient fuel and oxygen to the site of burning. Our tests therefore confirmed that intumescent system containing starch as a renewable carbonization agent can be used to produce superior PLA composites.

## Figures and Tables

**Table 1:** FR properties of PLA/APP and PLA/APP/ST composites.

No	Formulations	PLA wt %	APP %	ST %	LOI %	UL-94	Dripping
1	PLA	100	0	0	19.5	Failed	Y/Y
2	PLA/APP10	90	10	0	24.4	V-2	Y/Y
3	PLA/APP15	85	15	0	28.5	V-1	N/Y
4	PLA/APP20	80	20	0	31.9	V-0	N/Y
5	PLA/APP20/ST3	77	20	3	34.5	V-0	N/N
6	PLA/APP20/ST5	75	20	5	36.2	V-0	N/N
7	PLA/APP20/ST7	73	20	7	37.3	V-0	N/N

FR = Flame retardant, PLA = Polylactic acid, APP = Ammonium polyphosphate, ST = Starch, LOI = Limiting oxygen index, N/Y corresponds to NO/YES for dripping during the first/second flame application.

**Table 2:** Cone calorimetry data for pure PLA, PLA/APP, and PLA/APP/ST composites.

No.	Formulation	TTI (s)	PHRR (kW m^−2^)	THR (MJ m^−2^)	Residual Mass (%)
1	PLA	41 ± 1.3	570 ± 4	58 ± 0.11	0 ± 0.00
2	PLA/APP10	48 ± 1.7	461 ± 6	46 ± 0.18	14 ± 0.03
3	PLA/APP15	53 ± 2.2	378 ± 7	44 ± 0.32	17 ± 0.06
4	PLA/APP20	58 ± 1.5	337 ± 4	38 ± 0.23	22 ± 0.08
5	PLA/APP20/ST3	63 ± 2.1	212 ± 6	28 ± 0.19	26 ± 0.05
6	PLA/APP20/ST5	67 ± 1.4	200 ± 5	26 ± 0.13	37 ± 0.04
7	PLA/APP20/ST7	77 ± 1.8	192 ± 3	24 ± 0.28	43 ± 0.06

TTI = time to ignition; PHHR = peak heat release rate; THR = total heat release.

**Table 3:** Thermogravimetric analysis of PLA/APP and PLA/APP/ST composites.

No	Formulations	T_5_ (°C)	T_50_ (°C)	T_max_ (°C)	Residue at 700 °C (wt %)
1	PLA	325	372	377	0.00
2	PLA/APP10	320	374	379	5.90
3	PLA/APP15	346	376	378	8.16
4	PLA/APP20	358	376	378	9.21
5	PLA/APP20/ST3	365	380	379	13.34
6	PLA/APP20/ST5	371	381	380	15.32
7	PLA/APP20/ST7	373	383	380	19.30

T_5_ = 5% weight loss, T_50_ = 50% weight loss, T_max_ = Maximum rate of weight loss.

**Table 4:** Mechanical properties of PLA, PLA/APP, and PLA/APP/ST composites.

Formulations	Tensile Strength ± (MPa)	Elongation at Break ± (%)	Young’s Modulus ± (MPa)
PLA	69.19 ± 3	2.49 ± 0.2	4695.43 ± 21
PLA/APP10	47.86 ± 1	2.35 ± 0.4	4146.65 ± 18
PLA/APP15	46.12 ± 2	2.03 ± 0.3	4087.90 ± 17
PLA/APP20	45.62 ± 2	1.98 ± 0.1	3822.11 ± 15
PLA/APP20/ST3	43.93 ± 2	1.94 ± 0.1	3750.73 ± 13
PLA/APP20/ST5	39.30 ± 1	1.87 ± 0.1	2870.04 ± 14
PLA/APP20/ST7	38.41 ± 1	1.66 ± 0.1	2522.32 ± 11
